# DNA methylation map in circulating leukocytes mirrors subcutaneous adipose tissue methylation pattern: a genome-wide analysis from non-obese and obese patients

**DOI:** 10.1038/srep41903

**Published:** 2017-02-17

**Authors:** A. B. Crujeiras, A. Diaz-Lagares, J. Sandoval, F. I. Milagro, S. Navas-Carretero, M. C. Carreira, A. Gomez, D. Hervas, M. P. Monteiro, F. F. Casanueva, M. Esteller, J. A. Martinez

**Affiliations:** 1 Cancer Epigenetics and Biology Program (PEBC), Bellvitge Biomedical Research Institute (IDIBELL), L’Hospitalet, Catalonia, Spain; 2 Laboratory of Molecular and Cellular Endocrinology, Instituto de Investigación Sanitaria (IDIS), Complejo Hospitalario Universitario de Santiago (CHUS) and Santiago de Compostela University (USC), Santiago de Compostela, Spain; 3 CIBER Fisiopatología de la Obesidad y Nutricion (CIBERobn), Madrid, Spain; 4 Laboratory of Personalized Medicine, Epigenomics Unit, Medical Research Institute La Fe, Valencia, Spain; 5 Dpt. Nutrition, Food Sciences and Physiology, University of Navarra (UNAV) and IDISNA, Pamplona, Spain; 6 Biostatistics Unit, Medical Research Institute La Fe, Valencia, Spain; 7 Clinical and Experimental Endocrinology, Dept of Anatomy, Multidisciplinary Unit for Biomedical Research (UMIB), ICBAS, University of Porto, Portugal; 8 Institucio Catalana de Recerca i Estudis Avançats (ICREA); Passeig de Lluís Companys, 23, Barcelona, Catalonia, Spain; 9 Department of Physiological Sciences II, School of Medicine, University of Barcelona, Barcelona, Catalonia, Spain

## Abstract

The characterization of the epigenetic changes within the obesity-related adipose tissue will provide new insights to understand this metabolic disorder, but adipose tissue is not easy to sample in population-based studies. We aimed to evaluate the capacity of circulating leukocytes to reflect the adipose tissue-specific DNA methylation status of obesity susceptibility. DNA samples isolated from subcutaneous adipose tissue and circulating leukocytes were hybridized in the Infinium HumanMethylation 450 BeadChip. Data were compared between samples from obese (n = 45) and non-obese (n = 8–10) patients by Wilcoxon-rank test, unadjusted for cell type distributions. A global hypomethylation of the differentially methylated CpG sites (DMCpGs) was observed in the obese subcutaneous adipose tissue and leukocytes. The overlap analysis yielded a number of genes mapped by the common DMCpGs that were identified to reflect the obesity state in the leukocytes. Specifically, the methylation levels of FGFRL1, NCAPH2, PNKD and SMAD3 exhibited excellent and statistically significant efficiencies in the discrimination of obesity from non-obesity status (AUC > 0.80; p < 0.05) and a great correlation between both tissues. Therefore, the current study provided new and valuable DNA methylation biomarkers of obesity-related adipose tissue pathogenesis through peripheral blood analysis, an easily accessible and minimally invasive biological material instead of adipose tissue.

The prevalence of obesity and its associated disorders is increasing extremely rapidly worldwide and they are currently considered to be pandemic non-communicable diseases and a major challenge for the healthcare system^1^,^2^. Many metabolic alterations, such as obesity, are due to the interplay between environmental, lifestyle and genetic factors^3^. It is well known that physical inactivity and unhealthy dietary patterns exert major influences on metabolic syndrome, diabetes and obesity^4^. However, despite intensive genetic research into these alterations, the basic mechanisms and pathogenesis of obesity are still poorly understood. In this regard, emerging evidence suggests that epigenetics represents one link between environmental factors and the greater predisposition to develop obesity and its associated comorbidities^5^,^6^,^7^,^8^.

Epigenetic markers might explain the link between lifestyle and the risk of disease and have been proposed to be sensitive biomarkers and potential therapeutic targets for disease management that could contribute to personalized medicine^9^. The epigenetic machinery involves several levels of regulation, such as DNA methylation, post-translational histone modifications, nucleosome positioning and non-coding RNAs^10^. Among the epigenetic mechanisms, DNA methylation is the best-known epigenetic marker and has been proposed as a new generation of biomarkers with great diagnostic and prognostic promise for clinicians^10^. Regarding obesity susceptibility, several studies have investigated methylation sites in or near known candidate genes and provided evidences that obesity is associated with altered epigenetic regulation of a number of metabolically important genes^5^,^11^,^12^. Moreover, with the recent development of genome-wide methods for quantifying site-specific DNA methylation, studies investigating associations across a large number of genes and CpGs are being conducted with leukocytes or adipose tissue^5^,^11^,^12^. These approaches identified obesity-associated differentially methylated sites that are enriched in obesity candidate genes and in other genes with a wide diversity of other functions or even unknown properties related to obesity or adipose tissue biology^5^,^13^,^14^.

Epigenetic changes may be more tissue specific, and obesity pathophysiology is strongly associated with changes in the physiological functions of adipose tissue that lead to adipose tissue dysregulation^15^. This fact represents a huge challenge to the search for obesity-associated epigenetic biomarkers for the diagnosis and management of the disease as adipose tissue is inaccessible without surgery. In lieu of adipose tissue, epigenetic markers might be detectable in easily accessible samples, such as peripheral blood. In the obesity field, a very recently published study provided evidence of an inverse correlation between the DNA methylation of HIF3A in blood leukocytes and its expression in adipose tissue, which suggests that whole blood can be used to identify robust and biologically relevant epigenetic variation related to BMI^14^. Additionally, it has further been demonstrated in a genome-wide DNA methylation study of monozygotic twins discordant for obesity and liver fat that blood epigenetic profiling has great potential for better characterizing the obesity phenotype and identifying subjects who are most at risk for developing metabolic complications^16^. A recent, large, prospective, case-control study demonstrated that differences in DNA methylation in peripheral blood predict future type 2 diabetes incidence^17^. Leukocytes were also engaged to evaluate the DNA methylation of the displacement loop region of mitochondrial genome in obesity and was associated with insulin resistance^18^. Moreover, the influence of physical activity^19^, dietary factors^20^, gender and race/ethnicity^21^ on the DNA methylation of peripheral blood cells has been demonstrated. Additionally, it has been demonstrated that responses to weight loss treatment can be influenced and predicted by the DNA methylation status of leukocytes prior to treatment^22^,^23^.

The aim of the current study was to evaluate the capacity of circulating leukocytes to reflect the DNA methylation status of adipose tissue in an attempt to provide better insight in adipose tissue-specific epigenetic signatures of obesity susceptibility in easily accessible samples, such as peripheral blood instead of adipose tissue.

## Results

### Global pattern of subcutaneous adipose tissue DNA methylation in obese and non-obese subjects

**Figure 1:**
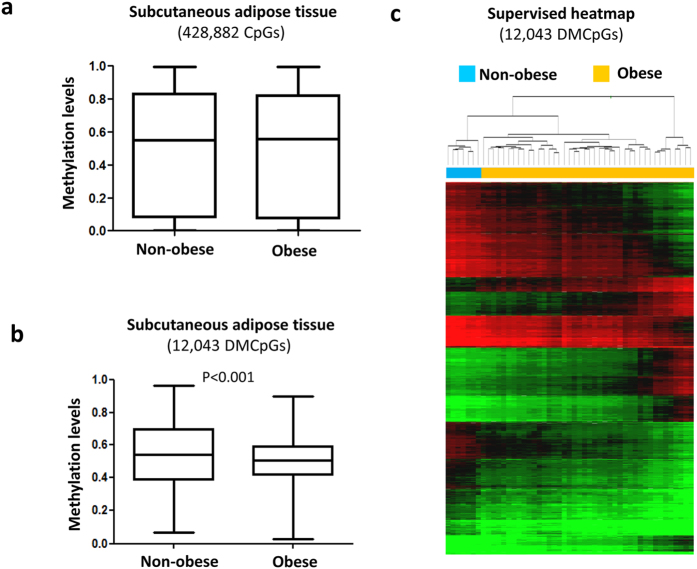
Profile of DNA methylation according to obesity state in adipose tissue. Differences in the global methylation levels of the overall valid CpGs between the obese and non-obese subcutaneous adipose tissue samples (**a**). Global differences in the methylation levels of the 12,043 differentially methylated CpGs (DMCpGs) identified by 450-k array analysis (**b**). Supervised clustering of the 12,043 CpGs that were found to be differentially methylated between the obese and non-obese groups (**c**). P-values were computed using wilcoxon rank sum test.

We performed a genome-wide DNA methylation analysis to characterize the global methylation status in obesity. We found that the average global levels subcutaneous adipose tissue DNA methylation (Fig. 1a) were similar between the obese and non-obese subjects. When subsequent analyses were performed to compare each of the CpG sites individually, 12,043 CpG sites were found to be significantly differentially methylated in the subcutaneous adipose tissue of obese compared with non-obese subjects based on the application of a false discovery rate (FDR) of <0.05 and a difference between groups of delta β ≥ 10% (see Supplementary Table S3). A quantile-quantile (QQ) plot representing the expected and observed p-values (lambda value = 3.9) is shown in Supplementary Figure S1a.

**Figure 2:**
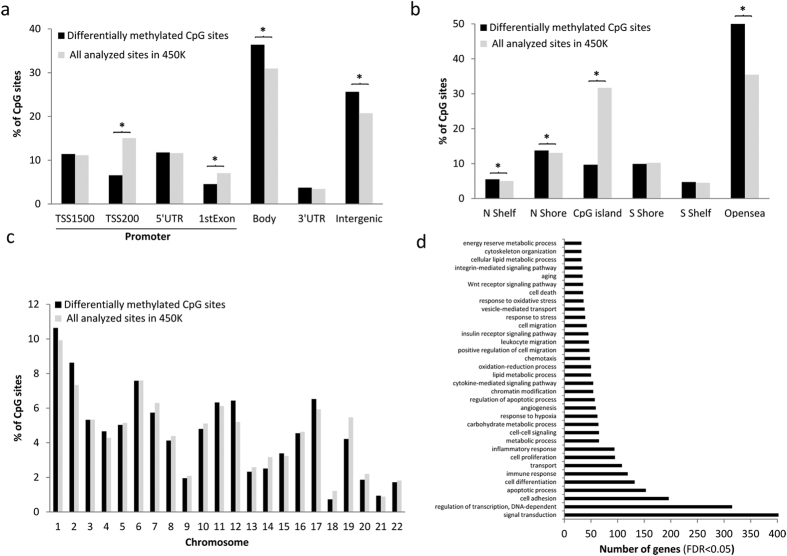
Characteristics of the candidate differentially methylated CpGs (DMCpGs). Genomic distributions of the differentially methylated CpG sites and their respective locations regarding the broader CpG context (**a**), the gene region (**b**) and chromosome distribution (**c**). Summary of the gene ontology (GO) analysis of the biological process categories for the 4,481 genes represented by the 12,043 differentially methylated CpG sites (**d**). *Significant differences (p < 0.05) in the distributions of differentially methylated CpG sites compared with all analyzed sites on the Infinium HumanMethylation450 BeadChip, by chi-square test.

Among the differentially methylated CpGs (DMCpGs), the obese subcutaneous adipose tissue exhibited lower methylation levels than the non-obese subcutaneous adipose tissue (Fig. 1b). The highest CpG frequencies in the obese samples were in the range of 0.40 to 0.60 (β-values), whereas the non-obese samples exhibited β-values greater than 0.60. These results suggest that adipose tissue from obese patients is characterized by a greater hypomethylation level. The identified CpG sites corresponded to 4,481 unique genes and they were able to separate both groups of samples using a hierarchical cluster approach (Fig. 1c). Compared across all analyzed sites in the Infinium HumanMethylation450 BeadChip, the DMCpGs of the subcutaneous adipose tissue in obese patients respect to non-obese subjects were underrepresented in the TSS200 and 1st exon and overrepresented in the body and intergenic regions (Fig. 2a). Regarding the CpG context, the DMCpGs were overrepresented in shelf, shore and open sea positions, whereas a statistically significant underrepresentation was observed in CpG island regions (Fig. 2b). Moreover, the distribution of the DMCpGs were primarily found on chromosomes 1, 2, 12 and 19 compared with all analyzed sites (Fig. 2c). Noteworthy, a gene ontology (GO) analysis showed that the identified obesity-related CpGs (4,481 genes) were enriched in biological functions related to the adipose tissue, such as cell adhesion and differentiation, immune response, transport, inflammatory responses, metabolic processes and response to hypoxia (Fig. 2d).

### Mirroring the subcutaneous adipose tissue DNA methylation map in leukocytes from obese and non-obese subjects

**Figure 3:**
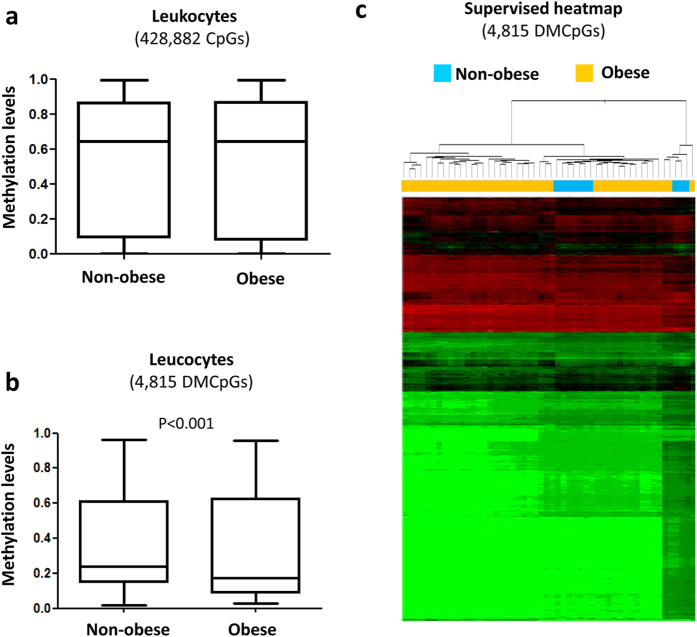
Profile of DNA methylation depending on obesity state in leukocytes. Differences in the global methylation levels of the overall valid CpGs between the obese and non-obese leukocyte samples (**a**). Global differences in the methylation levels of the 4,815 differentially methylated CpGs identified by 450-k array analysis (**b**). Supervised clustering of the 4815 CpGs that were found to be differentially methylated between the obese and non-obese group (**c**). P-values were computed using wilcoxon rank sum test.

Like in adipose tissue, the global DNA methylation levels in the leukocytes were similar between the non-obese and obese patients (Fig. 3a). When the CpGs were analyzed individually, 4,815 CpGs were found to be differentially methylated in the obese patients compared with the non-obese patients based on the application of a false discovery rate (FDR) of <0.05 and a difference between groups of delta β > 5% (Fig. 3b and see Supplementary Table S4). A quantile-quantile (QQ) plot representing the expected and observed p-values (lambda value = 3.2) is shown in Supplementary Figure S1b.

**Figure 4:**
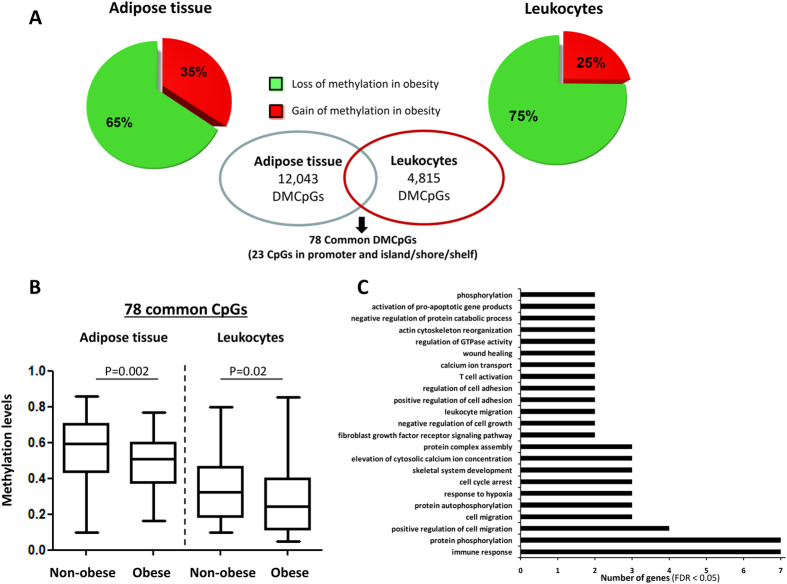
Comparison of the differential methylation profiles associated with obesity between the adipose tissue and leukocyte samples. A Venn diagram was created to obtain the common differentially methylated CpGs (DMCpGs) (**A**). Global differences in the methylation levels of the 78 common differentially methylated CpGs identified by the 450-k array analysis (**B**). Summary of the gene ontology (GO) analysis of the biological process categories for the differentially methylated genes represented by the CpG sites located in the promoter regions and islands (**C**). P-values were computed using wilcoxon rank sum test.

Although a lower percentage of DMCpGs was observed in the leukocytes than in the subcutaneous adipose tissue, the direction of the overall methylation was similar with a higher percentage of methylation lost in both tissues from the obese patients (Fig. 3b) and the identified DMCpGs were able to separate both groups of samples using a hierarchical cluster approach (Fig. 3c). Next, to identify the CpGs in leukocytes that were able to reflect the obesity state of the adipose tissue, a Venn diagram was created using the DMCpGs that were identified in the adipose tissue and leukocytes. This analysis yielded 176 common CpGs from which 78 CpGs that represented 68 unique genes exhibited the same methylation direction in both tissues. Among these CpGs, 13 exhibited high methylation levels, and 66 exhibited low methylation levels in the obese patients relative to the non-obese patients (Fig. 4A and Supplementary Table S5). Indeed, the 78 common CpGs exhibited globally lower methylation levels in the obese adipose tissue and leukocytes compared with the non-obese samples (Fig. 4B). A GO analysis was performed to test whether some molecular functions and biological processes were significantly associated with the 68 common genes that exhibited differences in DNA methylation status between both groups. Among the significantly enriched biological processes, the top overrepresented categories were immune response, cell migration, cell adhesion, response to hypoxia, cell cycle arrest and transdifferentiation (Fig. 4C).

In order to assess the biological relevance, the CpG sites representing promoter regions (TSS1500, TSS200, 5′UTR and 1^st^ exon) in the islands and shores were selected. Table 1 reports the 23 CpG sites with greatest differences between the obese and non-obese patients in both the adipose tissue and leukocytes ranked by gene name. Among these genes, FGFRL1, LMF2, NCAPHR, PODXL, PIP5K1A, PNKD, PTPRCAP, RSRC2, KNTC1, SMAD3 and WDR45L (corresponding to 9 CpG sites) were identified as genes that potentially reflect the obesity state in the leukocytes because they exhibited differences of similar magnitude (differences greater than 5%) in terms of the β-values (Table 2). Gender does not exhibited statistically significant influence to the methylation levels of the studied genes and the association between the methylation levels of the identified genes was maintained when BMI was included as continuous variable adjusted for gender (data not shown). Moreover, after literature search, these identified genes containing obesity-related DMCpGs were apparently involved in important process associated with adipose tissue homeostasis, such as cell proliferation and differentiation, senescence, network of inflammation, cell-cell adhesion and mitochondrial function as has been previously reported (Table 2).

Because DNA methylation affect transcription, the mRNA expression of the identified genes was examined, based on reported subcutaneous adipose tissue gene expression differences between obese and non-obese patients^24^. Relevantly, an inverse correlation was observed between methylation and gene expression of 6 genes: PNKD, PODXL, PTPRCAP, SMAD3 and WDR45L as well as a trend for statistical correlation in FGFRL1 (Table 2). Moreover, we found correlations between BMI and the β-values of the CpGs in the subcutaneous adipose tissue mapped to, PNKD (cg22712983; r = 0.35; p = 0.014), PODXL (cg02051077; r = −0.33; p = 0.021), PTPRCAP (cg02874905; r = −0.36; p = 0.011), and SMAD3 (cg07576222; r = −0.28; p = 0.049). Additionally, FGFRL1 (cg25932599; r = −0.27; p = 0.059) and PIPK1A (cg06936779; r = −0.26; p = 0.067) showed a trend for statistical correlation with BMI.

**Figure 5:**
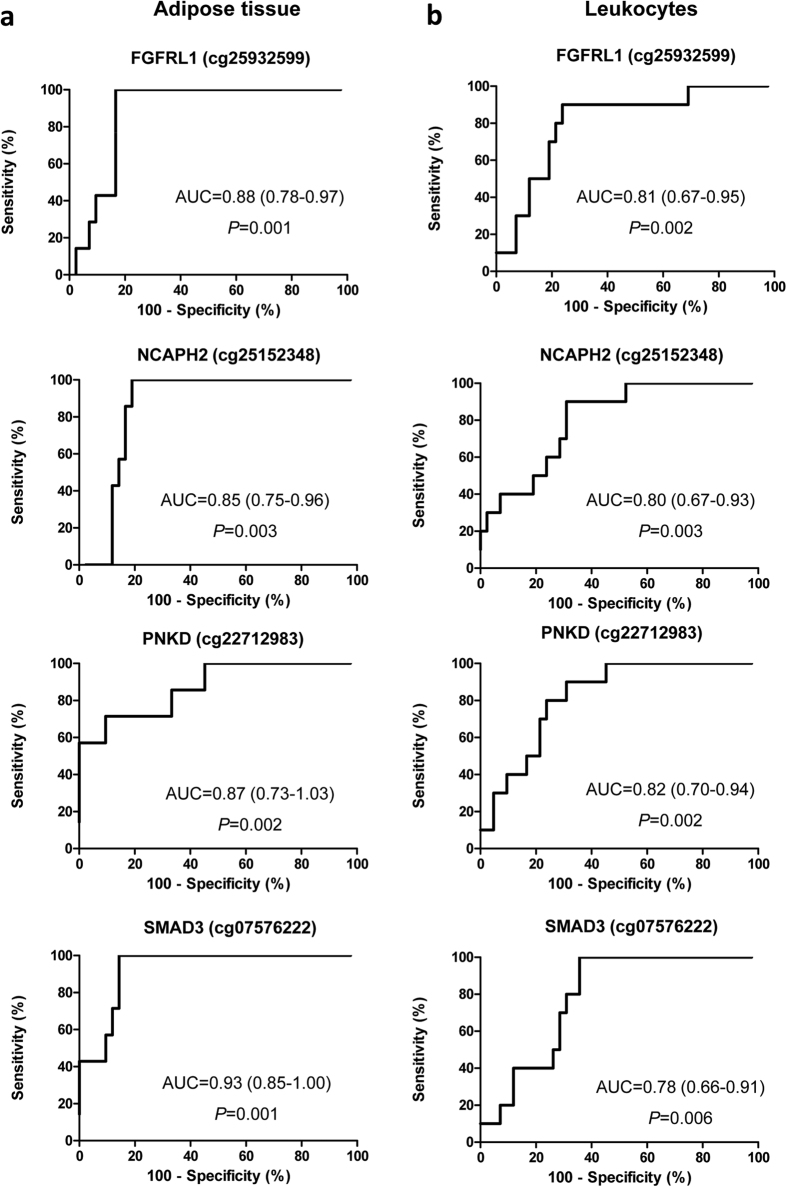
Receiver operating characteristic (ROC) curves for the methylation levels of the obesity-related differentially methylated CpGs. The abilities to discriminate the obese from the non-obese samples of adipose tissue (**a**) and leukocytes (**b**).

To further analyze the diagnostic power of the identified DMCpGs, receiver operating characteristic (ROC) curves were performed (Fig. 5). Relevantly, the areas under the ROC curves (AUCs) for FGFRL1, NCAPH2, PNKD, and SMAD3 evidenced excellent and statistically significant efficiencies in discriminating obesity from non-obesity in both the subcutaneous adipose tissue (Fig. 5a) and leukocytes (Fig. 5b). Moreover, we found a great correlation in the DNA methylation between the subcutaneous adipose tissue and leukocytes for FGFRL1 (r = 0.77; p < 0.001), NCAPH2 (r = 0.68; p < 0.001), PODXL (r = 0.70; p < 0.001) and SMAD3 (r = 0.74; p < 0.001).

## Discussion

The characterization of the epigenetic changes associated with obesity will provide new insights about the pathophysiological processes that are involved in this metabolic disorder and help us to develop better approaches for prevention and disease management, useful biomarkers and treatment strategies. In the current work, we used Illumina HumanMethylation 450k arrays to characterize the genome-wide DNA methylation profile associated with obesity in adipose tissue and to identify the CpG sites in circulating leukocytes that were differentially methylated relative to adipose tissue. The overlap analysis sought to identify DNA methylation markers that can be detected in an easily accessible biological source, i.e., blood, yielded 78 differentially methylated CpGs that exhibited changes in the same directions in the adipose tissue and the leukocytes. These 78 CpGs anoted to 68 unique genes. Nine of these 78 CpGs were located in promoters and islands and they mapped to 11 unique genes (FGFRL1, LMF2, NCAPHR, PODXL, PIP5K1A, PNKD, PTPRCAP, RSRC2, KNTC1, SMAD3 and WDR45L) which were identified as potential target genes that could reflect the obesity state in leukocytes because they exhibited the greatest differences between the obese and non-obese samples. These genes were involved in important processes associated with adipose tissue homeostasis, such as cell proliferation and differentiation, senescence, the network of inflammation, cell-cell adhesion and mitochondrial function. Moreover, the expression of FGFRL1, PNKD, PODXL, PTPRCAP, SMAD3 and WDR45L based on previously reported data correlated inversely with the methylation levels in the subcutaneous adipose tissue suggesting a potential epigenetic regulation associated to obesity.

Strong evidence that complex diseases, such as metabolic disorders, are under the influence of epigenetic modifications even in early life is opening up exciting new avenues for the identification of DNA methylation biomarkers associated with these disorders and the estimation of future disease risk^25^. Biomarkers are naturally occurring characteristics from which particular pathological processes or diseases can be identified or monitored. Biomarkers reflect past environmental exposures, predict disease onset and course and determine a patient’s response to therapy. Epigenetic changes represent these characteristics, and the majority of epigenetic biomarkers that have been discovered to date are based on the epigenetic marker of DNA methylation. DNA methylation biomarkers with diagnostic, prognostic and predictive powers are already in clinical trials and are used in clinical settings for some diseases such as cancer^26^.

Regarding obesity susceptibility, studies have investigated methylation sites in or near known candidate genes and across a large number of genes and CpGs with genome-wide methods. These approaches identify obesity-associated differentially methylated sites that are enriched in obesity candidate genes and in genes with a wide diversity of other functions or even unknown properties related to obesity or adipose tissue functions^5^,^11^. Thus, specific patterns of DNA methylation have been found to be associated with obesity itself^14^,^27^,^28^, with BMI^14^,^29^ and with responses to obesity therapy^22^,^23^,^30^,^31^,^32^ in specific candidate gene and genome-wide DNA methylation analyses in leukocytes and/or adipose tissue. Recently, the methylome of isolated human fat cells from post-obese and BMI-matched never-obese women was also characterized, and a number of adipogenesis and lipid metabolism genes have been identified as epigenetically regulated^33^,^34^.

Because blood is easily accessible and is routinely sampled in clinical and large-scale studies, peripheral blood cells are the most frequently used source of DNA for epigenetic studies. However, epigenetic changes may be more tissue-specific, and the blood cell methylation profile may not necessarily reflect the epigenetic states in other tissues. Currently, a massive effort is directed at providing better insight into tissue-specific epigenetic signatures and their roles in disease development. In this regard, very recently, Rönn *et al*.^29^ demonstrated that epigenetic biomarkers in blood can mirror age-related epigenetic signatures in biologically relevant target tissues such as adipose tissue. Moreover, Dick and colleagues^14^ described the first systematic analysis of the association between variation in DNA methylation and body mass index. That work reported an inverse correlation between DNA methylation and HIF3A expression in adipose tissue and drew attention to the potential functional relevance of epigenetic variation at the identified locus. Relevantly, the DNA methylation profile of HIF3A was reflected in whole blood, which suggests that the assessment of DNA methylation in whole blood can identify robust and biologically relevant epigenetic variation that is related to BMI^14^. In the current research, we were unable to detect differences in methylation levels of HIF3A in subcutaneous adipose tissue nor in leukocytes, probably because differences in the study design, sample size, the threshold used for selection of candidates and even the location of subcutaneous adipose tissue sampling. However the current study adds new information to this issue. By comprehensively interrogating 485,000 CpG sites in samples of subcutaneous adipose tissue 12,043 CpG sites annoted to 4,481 unique genes were found to be differentially methylated between obese and non-obese samples. Among the genes associated to these DMCpGs, 12 genes (CHT11, ZBTB16, MAD1L1, E2F5, FGFRL1, CCDC92, ACSF3, MAML3, ASAP2, EHBP1L1, RBPMS, TBC1D16) were also observed in a previous study that compares subcutaneous adipose tissue of BMI-discordant monozygotic twin pairs, even though not in the same CpG loci for some of them^28^. PLEC1 and ITIH5 were also found to be inversely associated with obesity state in our study, similar to that reported by Rönn *et al*.^29^. Moreover, among the genes mapped by the DMCpGs revealed in the subcutaneous adipose tissue we found a significant number of defined obesity genes that were previously demonstrated to undergo DNA methylation changes after gastric bypass and weight loss^31^. As examples of these common genes were FTO, LEPR, PHACTR1, SLC22A8, NDUFS2, NCOR2, PRDM16 several homeobox genes (HOXA3, HOXC4, HOXD3, HOXD4, HOXB6, MSX1, EMX2OS, EMX2) and genes involved in epigenome regulation (HDAC4, HDAC3, HDAC9, DNMT3A).

Strikingly, by means of a stringent filtering we were able to identify an obesity-related epigenetic signature of adipose tissue in a biological source that is easily accessible, i.e., blood leukocytes. The identified epigenetic signature could be relevant to the personalized management of obesity because most of the associated genes are involved in pathways associated with adipose tissue function. Thus, the most overrepresented pathways of differential methylated CpG sites were related to the structural components of the cellular matrix, inflammation and adipogenesis. Among the identified differentially methylated genes, fibroblast growth factor receptor-like 1 (FGFRL1) has been postulated to be involved in metabolism signalling and insulin processing^35^ and it is an early indicator of adipogenesis^36^. Moreover, FGFRL1 exerts a negative effect on cell proliferation and a positive effect on cell differentiation^37^ and is involved in tumor growth^38^. Noteworthy, the body of this gene was found to be hypermethylated in the subcutaneous adipose tissue of BMI-discordant monozygotic twin pairs^28^. By contrast in the current study low methylation levels of its promoter and island was found in the obese patients, similar to previous report^29^. The condensin II gene NCAPH2 is a recently discovered gene that has been found to be involved in senescence^39^, which is a process that is increased in dysfunctional obese adipose tissue and is related to the development of metabolic disturbances^40^. The most relevant differentially methylated gene identified was SMAD family member 3 (SMAD3). This gene is a TGF-beta effector protein and is a miscellaneous factor of adipogenesis. The loss of SMAD3 results in the transformation of white adipose tissue (WAT) to a brown adipose tissue (BAT)-like/beige cell phenotype and thus increases basal energy expenditure and protects against high-fat diet-induced obesity and type 2 diabetes mellitus^41^. Therefore, the blockage of SMDA3 protects against obesity and type 2 diabetes mellitus^41^. The WAT of SMAD3^−^/^−^ mice exhibits decreased adiposity, and these mice also exhibit increased whole-body glucose uptake and insulin sensitivity and decreased expression of genes involved in fatty acid storage^42^. This effect is probably attributable to the modulation of WAT metabolism, which increases mitochondrial biogenesis. Regarding mitochondrial function, mutations of the identified paroxysmal nonkinesigenic dyskinesia (PNKD) gene are involved in the development of a disease induced by mitochondrial dysfunction^43^. Although there is currently no evidence regarding the involvement of this gene in adipose tissue function or the pathogenesis of obesity, the increased methylation levels observed in the adipose tissue and leukocytes of the obese patients compared with the non-obese patients in the current work suggest that this gene should be further functionally explored in terms of its role in the pathogenesis of the obese adipose tissue.

The strength and novelty of the current work are represented in the paired analyses of the adipose tissues and circulating leukocytes from the same patients. These analyses allowed us to select an epigenetic signature of the obesity-related adipose tissue phenotype with high accuracy based on a non-invasive and easy accessible source of DNA. The difference in sample size and age between the non-obese and obese patients could be a limitation of this study because the effects of age on obesity-related epigenetic markers need to be further determined. In fact, the association between age and the DNA methylation profile was previously reported^29^,^44^. Moreover, as several technical factors and environmental exposures are thought to cause aberrant DNA methylation, the effect of dietary factors and smoking, as well as the tissue heterogeneity and cell type, among others should also be taken into consideration in the differential methylation analysis by performing a multi-variate analysis. In the current study the Wilcoxon rank test was performed for the analysis instead of using linear regression models because the sample size to gain robustness. Moreover, the results were focused only in those CpG sites with a methylation pattern in leukocytes similar to that observed in adipose tissue and located in promoter and islands/shores regions. This further filtering out was performed in order to minimize the impossibility of controlling for potential confounder factors. The absolute differences in DNA methylation between the obese and non-obese patients were small in both the subcutaneous adipose tissue and the leukocytes, although these differences could have functional significances similar to those previously reported for human adipose tissue^45^,^46^,^47^,^48^, isolated fat cells^33^,^34^ and human skeletal muscle^49^,^50^. Despite these facts, the data obtained in the current study provide new and valuable DNA methylation biomarkers together with other previously reported data in adipose tissue. The proposed epigenetic biomarkers in the current study could help to elucidate the molecular mechanisms involved in adipose tissue pathogenesis associated with obesity that can be examined in an easily accessible biological material, i.e., blood. During the peer-review process of the current work, an independent study evaluating epigenome-wide profiling of DNA methylation in paired samples of adipose tissue and blood suggested that the magnitude of within-subject correlation between blood and adipose tissue does not guarantee the utility of surrogate epigenetic markers because the association is phenotype-specific^51^. Relevantly, the current work reports a number of CpG sites that has a similar pattern of methylation levels in both adipose tissue and leukocytes and both are associated with an obesity state.

Further prospective studies are needed to corroborate the suitability of the proposed obesity-related epigenetic signature in leukocytes for clinical applications that improve obesity understanding and management taking into account the tissue heterogeneity and cell type as well as the environmental factors that influence the DNA methylation pattern. In conclusion, the current results provide evidence that the blood DNA methylation profile has great potential for better characterizing the obesity phenotype and improving the early diagnosis of and effective personalized therapies for this metabolic disorder.

## Subjects and Methods

### Study participants

The DNA methylation statuses of a total of 108 DNA samples that were hybridized in the Infinium HumanMethylation 450 BeadChip were evaluated (see Supplementary table S1). A portion of these DNA samples were isolated from paired abdominal, periumbilical subcutaneous adipose tissue and leukocytes from 45 obese patients (n = 23 women and n = 22 men). Alternative DNA samples from non-obese patients were obtained from abdominal, periumbilical subcutaneous adipose tissue (n = 8; 4 women and 4 men) and from leukocytes (n = 10; 5 men and 5 women). The range of ages and BMIs of the obese patients were 20 to 83 years and 26.2 to 53.1 kg/m^2^, respectively. The ranges of ages and BMIs of the non-obese patients were 23 to 79 years and 20.1 to 24.3 kg/m^2^, respectively (see Supplementary table S1). All participants were European Caucasian and provided a medical history, physical examination and routine laboratory tests. Normal-weight patients reported no history of diabetes mellitus, high blood pressure, dyslipidemia or major body weight changes in the last 3 months and were healthy overall. Exclusion criteria for study enrolment were pregnancy, alcohol or drug abuse and chronic prescriptions of other medical drugs different of antidiabetic medication.

Written informed consent to participate in the trial was obtained before the start of the study in agreement with the Declaration of Helsinki, and the study was performed following national and European Union guidelines. The protocol of this study was approved by the Clinica Universidad de Navarra Ethics Committee.

BMI was calculated as the weight in kg divided by the squared height in meters and was categorized using the following World Health Organization (WHO) criteria: normal/under-weight, BMI < 25 kg/m^2^; overweight, 25 ≤ BMI < 30 kg/m^2^; and obese, BMI ≥ 30 kg/m^2^ 1. Then, patients were classified considering overweight and obese patients in the same group, as obese (BMI > 25 kg/m^2^) and non-obese (BMI ≤ 25 kg/m^2^) to evaluate the effect of excess body weight.

The obese subcutaneous adipose tissue was obtained by biopsy from the periumbilical area using a 6-mm Bergstrom needle (Stile AB, Sweden) under local anesthesia (1% lidocaine). The non-obese subcutaneous adipose tissue was collected from patients who underwent elective laparoscopic surgery for non-infectious and non-oncologic disease (see Supplementary Table S2) from the periumbilical area. Venous blood samples were drawn by venipunture after a 12-h overnight fast. The EDTA-plasma samples and leukocytes (all white blood cells) were separated from whole blood by centrifugation (3500 rpm, 5 °C, 15 min), and were frozen immediately at −80 °C in buffy-coat.

### DNA preparation and bisulfite conversion

DNA from fresh-frozen (FF) tissue and blood samples was isolated using a standard phenol-chloroform/proteinase-k protocol according to the respective manufacturer’s instructions with slight modifications. The isolation of genomic DNA from subcutaneous adipose tissue was performed using the Qiagen DNeasy Blood & Tissue Kit (Qiagen, Valencia, CA). Genomic DNA was isolated from leukocytes by using the MasturPure TM DNA purification kit (Epicentre Biotechnologies, Madison, WI, USA). The obtained DNA was treated with RNase A for 1 h at 45 °C. All DNA samples were quantified using the fluorometric method (Quan-iT PicoGreen DsDNA Assay, Life Technologies) and were assessed for purity using a NanoDrop (Thermo Scientific) with 260/280 and 260/230 ratio measurements. The integrity of the FF DNA was verified by electrophoresis in 1.3% agarose gel. FF DNA (600 ng) was processed using the EZ-96 DNA Methylation kit (Zymo Research Corp.) following the manufacturer’s recommendations for Infinium assays.

### DNA methylation analysis

#### Infinium Human Methylation 450 BeadChip array

Microarray-based DNA methylation analysis was conducted with the Infinium Human Methylation 450 BeadChip (450k array). DNA quality checks, bisulfite modification, hybridization, data normalization, statistical filtering, and β value calculations were performed as described elsewhere^52^. High-quality DNA samples obtained from subcutaneous adipose tissue and leukocytes were selected for bisulfite conversion (Zymo Research; EZ-96 DNA Methylation™ Kit) and hybridization to the Infinium Human Methylation 450 BeadChips (Illumina) following the Illumina Infinium HD methylation protocol. The DNA concentration of the quality control sample standards was measured using the PicoGreen method (Invitrogen) coupled with assessments of DNA purity based on the A260/A280 ratio (ranging between 1.75 and 1.95) and the A260/A230 ratio (ranging between 2.00 and 2.20). Analysis with 1% agarose gel electrophoresis permitted the exclusion of samples with possible DNA fragmentation or RNA contamination. The Infinium Human Methylation 450 BeadChip provides coverage of >450,000 CpG sites targeting nearly all of the RefSeq genes (>99%)^52^. The chips were designed to cover the coding and non-coding genes without bias against those lacking CpG islands. The design further aimed to cover not only promoter regulatory regions but also CpGs across gene regions to include the 5′-untranslated regions (5′ UTRs), the first exons, the gene bodies and the 3′-untranslated regions (3′ UTRs).

A total of 600 ng from the high-quality DNA samples was bisulfite converted. Whole-genome amplification and hybridization were then performed on the BeadChip and followed by single-base extension and analysis on a HiScan SQ module (Illumina) to assess the cytosine methylation states. The annotation of the CGIs used the following categorization: 1) shore, for each of the 2-kb sequences flanking a CGI; 2) shelf, for each of the 2-kb sequences next to a shore; and 3) open sea, for DNA not included in any of the previous sequences or in CGIs^52^. The transcription start site 200 and the transcription start site 1500 indicate the regions either 200 or 1500 bp from the transcription start site, respectively.

### Gene expression assay

Gene expression levels of subcutaneous adipose tissue were obtained and analyzed from a previously published gene expression microarray dataset deposited at the Gene expression omnibus (GSE15524 GEO COHORT)^24^. Briefly, this study consisted in individualized analysis through expression profiling of 20,000 probes in 28 tissue samples evaluated in subcutaneous and omental adipose tissue obtained during surgical intervention in non-obese and obese patients. Patient samples from men and women of varying body size (lean to severely obese) were collected at the time of operation in the fasting state including.

### Statistical analyses

The methylation level of each cytosine was expressed as a β value that was calculated as the fluorescence intensity ratio of the methylated to the unmethylated alleles (Signal A and signal B are produced by two different bead types and reported in the same color) for infinium type I probes and (Signal A corresponds to the signal in the Red channel and signal B corresponds to the signal in the Green channel) for infinium type II probes. The β values ranged between 0 (unmethylated) and 1 (completely methylated) according to a combination of the Cy3 and Cy5 fluorescence intensities. Color balance adjustment and normalization were performed to normalize the samples between the two color channels using Genome Studio Illumina software (V2010.3). Genome Studio normalizes data using different internal controls that are present on the HumanMethylation 450 BeadChip. This software also normalized data depending on internal background probes^52^. β values with detection p-values > 0.01 were considered to fall below the minimum intensity and threshold, and these CpGs (40,710) were consequently removed from further analysis. Approximately 96% of the CpG islands were covered, along with regions proximal to the CpG islands (“CpG shores”) and the more distal CpG shelves. Additionally, the probes that were localized to the sex chromosomes (11,234 CpGs form Chromosome X and 416 from chromosome Y) and those CpGs that contain single nucleotide polymorphisms (SNPs) at the 10 bp 3′ end of the interrogating probe (1,179 CpGs) were filtered out. Additionally 72 rs (controls SNPs probes) and 3,343 ch (non-CpG loci) probes were also removed. The final valid CpGs for the study were 428,882.To identify consistent patterns of differentially methylated CpG sites (DMCpGs) between the obese and normal-weight adipose tissue and leukocyte samples, non-parametric Wilcoxon rank sum tests were performed. We performed Wilcoxon rank test for the analysis instead of using linear models or other methods able to adjust for confounding because of sample size. This test demonstrates quite robust results even for a small number of subjects. P values were adjusted for multiple comparisons using the false discovery rate (FDR) procedure of Benjamini and Hochberg. In this analysis, a FDR below 5% was considered statistically significant. Additionally, we applied a threshold for the significant sites based on the mean differences between groups with a minimum β value change of ±0.05. Hierarchical cluster analysis of the significant CpGs was performed using the heatmap function. All of the mentioned statistical analyses were performed using R software (version 3.2.0). The global methylation level was compared between obese and non-obese subjects by Wilcoxon rank test. The genomic distribution of the differentially methylated CpGs was compared with the distribution of the CpGs in all analyzed sites on the InfinumHumanMethylation 450 BeadChip. *P* values were computed using the chi-square test to determine over- or under-representation of the CpGs.

To estimate the enrichment in biological processes, a hypergeometric test was performed on the biological processes defined by gene ontology (GO)^52^. This analysis detected the significant over-representation of GO terms in one of the sets (i.e., list of identified genes) with respect to the other for the entire genome. GO terms with an adjusted p-value < 0.05 were considered significant.

A Venn diagram was created by crossing the DMCpGs that were identified in the adipose tissue with those identified in the leukocytes. The list of common CpGs was further filtered out by probes which map to multiple genomic locations^53^. From the complete list of the DMCpGs that exhibited the same methylation direction in leukocytes and adipose tissue, those DMCpGS located in promoter regions and CpG islands and shores were selected. Among the DMCpGs met these criteria that DMCpGs exhibiting differences of similar magnitude in terms of the β-values in leukocytes and subcutaneous adipose tissue were identified as signature of obesity state and were further analized.

With the SPSS version 17.0 software (SPSS Inc., Chicago, IL) for Windows XP (Microsoft, Redmond, WA), the potential association between BMI and DNA methylation levels (β-values) was evaluated using the Pearson coefficient test and a multivariate linear regression model was fitted to assess the association between the DNA methylation levels of the identified genes with BMI as a continuous variable adjusted for gender. Differences in DNA methylation levels and expression of the identified genes between groups were assessed by the non-parametric Mann Whitney U test. P ≤ 0.05 was considered statistically significant.

Additionally, the diagnostic efficiencies (percent correctly classified) of the candidate genes that were differentially methylated were calculated as the agreement percentages using receiver operating characteristic (ROC) curve analyses. These results are often interpreted as negligible efficiency (<20%), minimal efficiency (20–40%), moderate efficiency (41–60%), good efficiency (61–80%) and excellent efficiency (>80%).

## Additional Information

**How to cite this article**: Crujeiras, A. B. *et al*. DNA methylation map in circulating leukocytes mirrors subcutaneous adipose tissue methylation pattern: a genome-wide analysis from non-obese and obese patients. *Sci. Rep.* **7**, 41903; doi: 10.1038/srep41903 (2017).

**Publisher's note:** Springer Nature remains neutral with regard to jurisdictional claims in published maps and institutional affiliations.

## Supplementary Material

Supplementary Data

## Figures and Tables

**Table 1:** The twenty-three highest commonly differentially methylated CpGs in subcutaneous adipose tissue and leukocytes between obese (Ob) and non-obese (Nob) located in promoter and island and shore and sorted by gene name.

TargetID	CHR	Position	Gene name	Gene region	CpG context	Differences adipose tissue (Ob-Nob)	Differences leukocytes (Ob-Nob)
cg02379560	4	40859280	APBB2;APBB2;APBB2;APBB2;APBB2;APBB2;APBB2	Body;TSS200;TSS200;Body;TSS200;Body;TSS200	S_Shore	−0.11	−0.07
cg20251225	1	1609551	CDK11B;CDK11B;CDK11B;CDK11B;CDK11B;LOC728661;CDK11B	Body;Body;Body;Body;Body;5′UTR;5′UTR	S_Shore	−0.10	−0.06
cg15681239	3	38080203	DLEC1;DLEC1	TSS1500;TSS1500	N_Shore	−0.11	−0.06
cg25932599	4	1005201	FGFRL1;FGFRL1;FGFRL1	TSS1500;TSS1500;TSS1500	Island	−0.12	−0.11
cg15644324	1	42384647	HIVEP3;HIVEP3	TSS200;TSS200	S_Shore	0.13	0.07
cg00514575	5	180231155	MGAT1;MGAT1;MGAT1;MGAT1;MGAT1	TSS1500;5′UTR;TSS1500;5′UTR;TSS1500	S_Shore	−0.12	−0.06
cg11201447	8	128808063	MIR1204;PVT1	TSS200;Body	S_Shore	−0.10	−0.06
cg25152348	22	50946712	NCAPH2;LMF2;NCAPH2;NCAPH2;NCAPH2	1stExon;TSS1500;5′UTR;5′UTR;1stExon	Island	−0.10	−0.13
cg06936779	1	151171405	PIP5K1A;PIP5K1A;PIP5K1A;PIP5K1A;PIP5K1A;PIP5K1A; PIP5K1A;PIP5K1A	5′UTR;1stExon;5′UTR;1stExon;5′UTR;1stExon;1stExon;5′UTR	Island	−0.12	−0.09
cg18437077	7	100860918	PLOD3;PLOD3;ZNHIT1	1stExon;5′UTR;TSS200	Island	−0.11	−0.06
cg22712983	2	219187374	PNKD;PNKD	TSS1500;Body	N_Shore	0.12	0.10
cg02051077	7	131241343	PODXL;PODXL;PODXL;PODXL	5′UTR;1stExon;5′UTR;1stExon	Island	−0.11	−0.07
cg11381564	6	32808619	PSMB8;PSMB8	3′UTR;3′UTR	S_Shore	−0.14	−0.08
cg14010720	20	62168878	PTK6	TSS200	S_Shore	−0.13	−0.06
cg10542975	11	67205096	PTPRCAP;PTPRCAP	5′UTR;1stExon	S_Shore	−0.15	−0.06
cg02874908	11	67205113	PTPRCAP;PTPRCAP	5′UTR;1stExon	S_Shore	−0.12	−0.09
cg17858911	12	123012351	RSRC2;RSRC2;RSRC2;KNTC1	TSS1500;TSS1500;TSS1500;5′UTR	S_Shore	−0.12	−0.11
cg02996471	19	3178512	S1PR4	TSS1500	N_Shore	−0.12	−0.06
cg07496545	5	140683737	SLC25A2	TSS200	Island	0.12	0.05
cg07576222	15	67357975	SMAD3	TSS1500	Island	−0.11	−0.07
cg13104938	11	843956	TSPAN4;TSPAN4;TSPAN4;TSPAN4;TSPAN4;POLR2L; TSPAN4;TSPAN4	TSS1500;5′UTR;TSS200;5′UTR;5′UTR;TSS1500;5′UTR;5′UTR	S_Shore	0.11	0.06
cg23713156	17	80606235	WDR45L;WDR45L	5′UTR;1stExon	Island	−0.10	−0.09

**Table 2:** Relevant genes holding obesity-related differentially methylated CpGs (DMCpGs) and its association with gene expression.

TargetID	Gene name	Function	Methylation levels (UNAV COHORT)						Expression levels (GSE15524 GEO COHORT)					
			Obese		Non-obese		Delta	P-value	Obese		Non-obese		Delta	P-value
			Mean	SD	Mean	SD			Mean	SD	Mean	SD		
cg25932599	FGFRL1	Metabolism signaling and insulin processing^35^; cell proliferation and differentiation^37^	0.24	0.08	0.36	0.05	***−0.11***	**0.002**	2.80	2.93	0.70	0.49	***2.10***	0.067
cg25152348	NCAPH2	Senescence^39^	0.34	0.13	0.44	0.04	−0.10	**0.003**	2.14	0.38	2.15	1.34	−0.01	0.933
cg06936779	PIP5K1A	Involved in diverse cellular events through its product the phospholipid phophatidylinositol 4.5-bi-sphosphate (PIP2)^54^	0.23	0.07	0.36	0.04	−0.12	**<0.001**	0.20	0.14	0.26	0.19	−0.06	0.683
cg22712983	PNKD	Potentially involved in the mitochondrial function^43^	0.57	0.07	0.46	0.07	***0.12***	**0.002**	0.17	0.06	0.38	0.06	***−0.21***	**0.024**
cg02051077	PODXL	Proadhesive molecule involved in atherosclerosis^55^ and tumor growth^56^	0.27	0.07	0.38	0.02	***−0.11***	**0.001**	0.86	0.42	0.34	0.15	***0.53***	**0.024**
cg02874908	PTPRCAP	Regulatory network of inflammation^57^	0.73	0.07	0.85	0.02	***−0.14***	**<0.001**	0.42	0.29	0.14	0.05	***0.28***	**0.017**
cg17858911	RSRC2	Tumor suppressor involved in cell proliferation^58^	0.25	0.07	0.36	0.08	−0.12	**0.002**	12.49	6.56	13.05	4.71	−0.57	0.940
cg0757622	SMAD3	Miscellaneous factor of adipogenesis. transdiferentiation and glucose homeostasis^41^	0.30	0.07	0.41	0.04	***−0.11***	**<0.001**	0.14	0.06	0.02	0.01	***0.12***	**0.016**
cg23713156	WDR45L	No items found	0.23	0.08	0.34	0.04	***−0.10***	**0.005**	0.65	0.19	0.22	0.04	***0.43***	**0.012**

*P* value denotes statistical differences between obese and non-obese evaluated by Mann-Whitney U test.

## References

[b1] Apovian C. M. . Pharmacological management of obesity: an endocrine Society clinical practice guideline. J Clin Endocrinol Metab 100, 342–362 (2015). 25590212 10.1210/jc.2014-3415

[b2] Kelly T., Yang W., Chen C. S., Reynolds K. & He J. Global burden of obesity in 2005 and projections to 2030. Int J Obes (Lond) 32, 1431–1437 (2008). 18607383 10.1038/ijo.2008.102

[b3] Marti A., Martinez-Gonzalez M. A. & Martinez J. A. Interaction between genes and lifestyle factors on obesity. Proc Nutr Soc 67, 1–8 (2008). 18234126 10.1017/S002966510800596X

[b4] Leech R. M., McNaughton S. A. & Timperio A. The clustering of diet, physical activity and sedentary behavior in children and adolescents: a review. Int J Behav Nutr Phys Act 11, 4 (2014). 24450617 10.1186/1479-5868-11-4 PMC3904164

[b5] Milagro F. I., Mansego M. L., De Miguel C. & Martinez J. A. Dietary factors, epigenetic modifications and obesity outcomes: progresses and perspectives. Mol Aspects Med 34, 782–812 (2013). 22771541 10.1016/j.mam.2012.06.010

[b6] Crujeiras A. B. . Leptin resistance in obesity: An epigenetic landscape. Life sciences 140, 57–63 (2015). 25998029 10.1016/j.lfs.2015.05.003

[b7] Crujeiras A. B. & Casanueva F. F. Obesity and the reproductive system disorders: epigenetics as a potential bridge. Hum Reprod Update 21, 249–261 (2015). 25413685 10.1093/humupd/dmu060

[b8] Crujeiras A. B., Diaz-Lagares A., Carreira M. C., Amil M. & Casanueva F. F. Oxidative stress associated to dysfunctional adipose tissue: a potential link between obesity, type 2 diabetes mellitus and breast cancer. Free radic res 47, 243–256 (2013). 23409968 10.3109/10715762.2013.772604

[b9] Hamilton J. P. Epigenetics: principles and practice. Dig Dis 29, 130–135 (2011). 21734376 10.1159/000323874 PMC3134032

[b10] Portela A. & Esteller M. Epigenetic modifications and human disease. Nat Biotechnol 28, 1057–1068 (2010). 20944598 10.1038/nbt.1685

[b11] van Dijk S. J., Molloy P. L., Varinli H., Morrison J. L. & Muhlhausler B. S. Epigenetics and human obesity. Int J Obes (Lond) 39, 85–97 (2010). 10.1038/ijo.2014.34 24566855

[b12] Crujeiras A. B. & Diaz-Lagares A. In Epigenetic biomarkers and diagnostics (ed Garcia-Gimenez) Ch. 16, 313–329 (Elsevier, 2015).

[b13] Xu X. . A genome-wide methylation study on obesity: differential variability and differential methylation. Epigenetics 8, 522–533 (2013). 23644594 10.4161/epi.24506 PMC3741222

[b14] Dick K. J. . DNA methylation and body-mass index: a genome-wide analysis. Lancet 383, 1990–1998 (2014). 24630777 10.1016/S0140-6736(13)62674-4

[b15] Dizdar O. & Alyamac E. Obesity: an endocrine tumor? Med Hypotheses 63, 790–792 (2004). 15488649 10.1016/j.mehy.2004.01.046

[b16] Ollikainen M. . Genome-wide blood DNA methylation alterations at regulatory elements and heterochromatic regions in monozygotic twins discordant for obesity and liver fat. Clin Epigenetics 7, 39 (2015). 25866590 10.1186/s13148-015-0073-5 PMC4393626

[b17] Chambers J. C. . Epigenome-wide association of DNA methylation markers in peripheral blood from Indian Asians and Europeans with incident type 2 diabetes: a nested case-control study. *The lancet*. Diabetes & endocrinology 3, 526–534 (2015). 26095709 10.1016/S2213-8587(15)00127-8 PMC4724884

[b18] Zheng L. D. . Insulin resistance is associated with epigenetic and genetic regulation of mitochondrial DNA in obese humans. Clin Epigenetics 7, 60 (2015). 26110043 10.1186/s13148-015-0093-1 PMC4479353

[b19] Zhang F. F. . Physical activity and global genomic DNA methylation in a cancer-free population. Epigenetics 6, 293–299 (2011). 21178401 10.4161/epi.6.3.14378 PMC3092677

[b20] Zhang F. F. . Dietary patterns are associated with levels of global genomic DNA methylation in a cancer-free population. J Nutr 141, 1165–1171 (2011). 21525250 10.3945/jn.110.134536 PMC3095144

[b21] Zhang F. F. . Significant differences in global genomic DNA methylation by gender and race/ethnicity in peripheral blood. Epigenetics 6, 623–629 (2011). 21739720 10.4161/epi.6.5.15335 PMC3230547

[b22] Crujeiras A. B. . Association of weight regain with specific methylation levels in the NPY and POMC promoters in leukocytes of obese men: a translational study. Regul Pept 186, 1–6 (2013). 23831408 10.1016/j.regpep.2013.06.012

[b23] Milagro F. I. . A dual epigenomic approach for the search of obesity biomarkers: DNA methylation in relation to diet-induced weight loss. Faseb J 25, 1378–1389 (2011). 21209057 10.1096/fj.10-170365

[b24] MacLaren R. E., Cui W., Lu H., Simard S. & Cianflone K. Association of adipocyte genes with ASP expression: a microarray analysis of subcutaneous and omental adipose tissue in morbidly obese subjects. BMC medical genomics 3, 3 (2010). 20105310 10.1186/1755-8794-3-3 PMC2843642

[b25] Mikeska T. & Craig J. M. DNA methylation biomarkers: cancer and beyond. Genes (Basel) 5, 821–864 (2014). 25229548 10.3390/genes5030821 PMC4198933

[b26] Heyn H., Mendez-Gonzalez J. & Esteller M. Epigenetic profiling joins personalized cancer medicine. Expert Rev Mol Diagn 13, 473–479 (2013). 23782254 10.1586/erm.13.36

[b27] Milagro F. I. . CLOCK, PER2 and BMAL1 DNA methylation: association with obesity and metabolic syndrome characteristics and monounsaturated fat intake. Chronobiol Int 29, 1180–1194 (2012). 23003921 10.3109/07420528.2012.719967

[b28] Pietilainen K. H. . DNA methylation and gene expression patterns in adipose tissue differ significantly within young adult monozygotic BMI-discordant twin pairs. Int J Obes (Lond), doi: 10.1038/ijo.2015.221 (2015). 26499446

[b29] Ronn T. . Impact of age, BMI and HbA1c levels on the genome-wide DNA methylation and mRNA expression patterns in human adipose tissue and identification of epigenetic biomarkers in blood. Human mol genet 24, 3792–3813 (2015). 25861810 10.1093/hmg/ddv124

[b30] Bouchard L. . Differential epigenomic and transcriptomic responses in subcutaneous adipose tissue between low and high responders to caloric restriction. Am J Clin Nutr 91, 309–320 (2010). 19939982 10.3945/ajcn.2009.28085

[b31] Benton M. C. . An analysis of DNA methylation in human adipose tissue reveals differential modification of obesity genes before and after gastric bypass and weight loss. Genome Biol 16, 8 (2015). 25651499 10.1186/s13059-014-0569-x PMC4301800

[b32] Barres R. . Weight loss after gastric bypass surgery in human obesity remodels promoter methylation. Cell Rep 3, 1020–1027 (2013). 23583180 10.1016/j.celrep.2013.03.018

[b33] Dahlman I. . The fat cell epigenetic signature in post-obese women is characterized by global hypomethylation and differential DNA methylation of adipogenesis genes. Int J Obes (Lond) 39, 910–919 (2015). 25783037 10.1038/ijo.2015.31

[b34] Arner P. . The epigenetic signature of subcutaneous fat cells is linked to altered expression of genes implicated in lipid metabolism in obese women. Clin Epigenetics 7, 93 (2015). 26351548 10.1186/s13148-015-0126-9 PMC4562340

[b35] Silva P. N., Altamentova S. M., Kilkenny D. M. & Rocheleau J. V. Fibroblast growth factor receptor like-1 (FGFRL1) interacts with SHP-1 phosphatase at insulin secretory granules and induces beta-cell ERK1/2 protein activation. J Biol Chem 288, 17859–17870 (2013). 23640895 10.1074/jbc.M112.440677 PMC3682584

[b36] Widberg C. H. . Fibroblast growth factor receptor 1 is a key regulator of early adipogenic events in human preadipocytes. American journal of physiology. Endocrinology and metabolism Am J Physiol Endocrinol Metab 296, E121–131 (2009). 10.1152/ajpendo.90602.2008 18940940

[b37] Trueb B. Biology of FGFRL1, the fifth fibroblast growth factor receptor. Cell Mol Life Sci 68, 951–964 (2011). 21080029 10.1007/s00018-010-0576-3 PMC11115071

[b38] Agarwal D. . FGF receptor genes and breast cancer susceptibility: results from the Breast Cancer Association Consortium. Br J Cancer 110, 1088–1100 (2014). 24548884 10.1038/bjc.2013.769 PMC3929867

[b39] Yokoyama Y., Zhu H., Zhang R. & Noma K. A novel role for the condensin II complex in cellular senescence. Cell Cycle 14, 2160–2170 (2015). 26017022 10.1080/15384101.2015.1049778 PMC4613835

[b40] Tchkonia T. . Fat tissue, aging, and cellular senescence. Aging Cell 9, 667–684 (2010). 20701600 10.1111/j.1474-9726.2010.00608.x PMC2941545

[b41] Yadav H. . Protection from obesity and diabetes by blockade of TGF-beta/Smad3 signaling. Cell Metab 14, 67–79 (2011). 21723505 10.1016/j.cmet.2011.04.013 PMC3169298

[b42] Beaudoin M. S. . Novel effects of rosiglitazone on SMAD2 and SMAD3 signaling in white adipose tissue of diabetic rats. Obesity (Silver Spring) 22, 1632–1642 (2014). 24500776 10.1002/oby.20717

[b43] Ghezzi D. . A family with paroxysmal nonkinesigenic dyskinesias (PNKD): evidence of mitochondrial dysfunction. Eur J Paediatr Neurol 19, 64–68 (2015). 25453601 10.1016/j.ejpn.2014.10.003

[b44] Johansson A., Enroth S. & Gyllensten U. Continuous Aging of the Human DNA Methylome Throughout the Human Lifespan. PLoS One 8, e67378 (2013). 23826282 10.1371/journal.pone.0067378 PMC3695075

[b45] Ronn T. . A six months exercise intervention influences the genome-wide DNA methylation pattern in human adipose tissue. PLoS Genet 9, e1003572 (2013). 23825961 10.1371/journal.pgen.1003572 PMC3694844

[b46] Grundberg E. . Global analysis of DNA methylation variation in adipose tissue from twins reveals links to disease-associated variants in distal regulatory elements. Am J Hum Genet 93, 876–890 (2013). 24183450 10.1016/j.ajhg.2013.10.004 PMC3824131

[b47] Nilsson E. . Altered DNA methylation and differential expression of genes influencing metabolism and inflammation in adipose tissue from subjects with type 2 diabetes. Diabetes 63, 2962–2976 (2014). 24812430 10.2337/db13-1459

[b48] Guenard F. . Differential methylation in visceral adipose tissue of obese men discordant for metabolic disturbances. Physiol Genomics 46, 216–222 (2014). 24495915 10.1152/physiolgenomics.00160.2013

[b49] Barres R. . Acute exercise remodels promoter methylation in human skeletal muscle. Cell Metab 15, 405–411 (2012). 22405075 10.1016/j.cmet.2012.01.001

[b50] Ribel-Madsen R. . Genome-wide analysis of DNA methylation differences in muscle and fat from monozygotic twins discordant for type 2 diabetes. PLoS One 7, e51302 (2012). 23251491 10.1371/journal.pone.0051302 PMC3519577

[b51] Huang Y. T. . Epigenome-wide profiling of DNA methylation in paired samples of adipose tissue and blood. Epigenetics 11, 227–36 (2016). 26891033 10.1080/15592294.2016.1146853 PMC4854552

[b52] Sandoval J. . Validation of a DNA methylation microarray for 450,000 CpG sites in the human genome. Epigenetics 6, 692–702 (2011). 21593595 10.4161/epi.6.6.16196

[b53] Naeem H. . Reducing the risk of false discovery enabling identification of biologically significant genome-wide methylation status using the HumanMethylation450 array. BMC Genomics 15, 51 (2014). 24447442 10.1186/1471-2164-15-51 PMC3943510

[b54] Gonzales M. L. & Anderson R. A. Nuclear phosphoinositide kinases and inositol phospholipids. J Cell Biochem 97, 252–260 (2006). 16267839 10.1002/jcb.20655

[b55] Li X., Yao N., Zhang J. & Liu Z. MicroRNA-125b is involved in atherosclerosis obliterans *in vitro* by targeting podocalyxin. Mol Med Rep 12, 561–568 (2015). 25738314 10.3892/mmr.2015.3384

[b56] Snyder K. A. . Podocalyxin enhances breast tumor growth and metastasis and is a target for monoclonal antibody therapy. Breast Cancer Res 17, 46 (2015). 25887862 10.1186/s13058-015-0562-7 PMC4423095

[b57] Saban R. . Regulatory network of inflammation downstream of proteinase-activated receptors. BMC Physiol 7, 3 (2007). 17397547 10.1186/1472-6793-7-3 PMC1853107

[b58] Kurehara H. . A novel gene, RSRC2, inhibits cell proliferation and affects survival in esophageal cancer patients. Int J Oncol 30, 421–428 (2007). 17203224

